# Social and geographical factors affecting access to treatment of lung cancer

**DOI:** 10.1038/sj.bjc.6605257

**Published:** 2009-08-18

**Authors:** S M Crawford, V Sauerzapf, R Haynes, H Zhao, D Forman, A P Jones

**Affiliations:** 1 Airedale General Hospital, Skipton Road, Steeton, Keighley, West Yorkshire BD20 6TD, UK; 2 School of Environmental Sciences, University of East Anglia, Norwich, Norfolk NR4 7TJ, UK; 3 Respiratory and Systemic Infection Department, Centre for Infections, Health Protection Agency, 61 Colindale Avenue, London NW9 5EQ UK; 4 Centre for Epidemiology and Biostatistics, University of Leeds and Northern and Yorkshire Cancer Registry and Information Service, St James's Institute of Oncology, St James's University Hospital, Leeds, LS9 7TF, UK

**Keywords:** lung cancer, socioeconomic factors, distance, travel, thoracic surgery, histopathology

## Abstract

**Background:**

UK residents' healthcare is free of charge but uptake varies. Cancer survival is inferior to that of other Western European countries. We have used cancer registry data to assess factors associated with access to diagnosis and treatment of lung cancer in northern England.

**Method:**

We assigned 34 923 lung cancer patients diagnosed between 1994 and 2002 to quartiles for the deprivation score associated with their postcode and for the travel time to the relevant healthcare facility. Odds ratios, adjusted for age and sex, for undergoing interventions were calculated relative to the least deprived quartile living closest to the facility. The odds ratio for receiving chemotherapy for small-cell lung cancer (SCLC) was calculated according to the type of hospital where it was diagnosed.

**Results:**

The odds ratio for attainment of a histological diagnosis for the least deprived/furthest residence group was 0.83 (95% confidence 0.70–0.97) for the most deprived/nearest residence group was 0.74(0.62–0.87) and for the most deprived/furthest residence group it was 0.61 (0.49–0.75). The corresponding odds ratios for receipt of any active treatment were 0.93 (0.80–1.07), 0.74 (0.64–0.86), and 0.55 (0.46–0.67). The odds ratios for receipt of chemotherapy for SCLC were 1.27 (0.89–1.82), 1.21 (0.85–1.74) and 0.81 (0.52–1.28). Odds ratios for undergoing surgery for non-small cell lung cancer using (1) travel time to diagnosing hospital were 0.88 (0.70–1.11), 0.74 (0.59–0.94) and 0.60 (0.44–0.84). Using (2) travel time to a thoracic surgery facility they were 0.83 (0.65–1.06), 0.70 (0.55–0.89) and 0.55 (0.49–0.76).

**Conclusion:**

Living in a deprived locality reduces the likelihood of undergoing definitive management for lung cancer with the exception of chemotherapy for SCLC. This is amplified by travel time to services.

The Eurocare comparisons of cancer survival among European populations have consistently shown that the constituent countries of the United Kingdom have inferior survival compared with peer group countries for many sites of cancer even though the National Health Service (NHS) provides diagnostic and treatment services free of charge. This is notably true for lung cancer as is confirmed by the most recent data (Berrino *et al*, 2007). Detailed studies of the UK service are required to determine how this may arise.

Moreover, within the United Kingdom survival has been shown to be generally poorer for those from more deprived areas. For the majority of adult tumours a significantly greater proportion of patients from the most affluent areas survive to 5 years after diagnosis than those from disadvantaged neighbourhoods. It has been suggested that this is associated with a combination of factors acting to the disadvantage of those from less affluent areas. These include presence of comorbidities precluding aggressive treatments, patient delay in seeking medical advice and delay in referral acting through later stage of disease at diagnosis, and differential access to specialist treatment. However, the strength of association between these factors and survival varies between studies and tumour site (Woods *et al*, 2006). In the case of lung cancer there is evidence that patients from more deprived areas of the United Kingdom are significantly less likely to receive active treatment in general (Jack *et al*, 2006) and chemotherapy in particular (Campbell *et al*, 2002; Patel *et al*, 2007). Similarly Earle *et al* (2000) writing at a time when chemotherapy for non-small cell lung cancer was not standard management in the United Kingdom, found that higher socioeconomic status patients in the USA were more likely to receive this treatment. Previous workers have also shown that receipt of treatment can be associated with the specialism of the doctor first seen at hospital (Jack *et al*, 2006).

The work reported here was undertaken as part of a larger study examining the effect of travel time to health services on survival and treatment for five common cancers using data supplied by the Northern and Yorkshire Cancer Registry and Information Service (NYCRIS). It gave the opportunity to study the separate influence of deprivation and geographic access to specialist services and their influence on treatment received for lung cancer in an area of England showing a wide range of both deprivation and geographic access to health services.

In the United Kingdom, NHS outpatients' access to all services is through their general practitioner (GP) with whom they will normally have initial contact except when emergency services are required. Direct access to hospital-based specialists is not available. General hospitals provide a wide variety of services but certain disciplines have been concentrated in specialist centres. In the context of lung cancer, thoracic surgery and radiotherapy were centralised more than 45 years ago; this policy was endorsed in England, within the national Cancer Plan (The NHS Cancer Plan, 2000).

Histological confirmation of lung cancer diagnosis is the baseline point in management and the extent of this previously showed differences between the UK and The Netherlands (Crawford and Atherton, 1994). In this analysis, we investigate the influence of social and geographical factors on the attainment of histological diagnosis and on receipt of any form of active treatment (surgery, chemotherapy or radiotherapy). We further investigate their influence on receipt of the definitive optimal treatment, surgery in the case of non-small cell lung tumours and chemotherapy in the case of small-cell cancer. The indicators we have independently chosen are also those used in the National Lung Cancer Audit (2007).

## Patients and methods

Records of lung cancer registrations held by the NYCRIS during the period 1994–2002 were used. After excluding mesothelioma, atypical pathology types and cases only diagnosed postmortem a total of 34 923 patient records were available for analysis.

The process of developing a database appropriate for geographical analyses from the Registry records has been described elsewhere (Jones *et al*, 2008a). This database included the histological diagnosis obtained and the treatment or treatments delivered in the period up to 6 months, but usually shorter, following diagnosis. Car travel times from the patient's residence to healthcare providers were estimated in a geographical information system (ArcGIS 9.2) using the shortest road route and average driving speeds along specific classes of roads. An independent survey of 475 patients attending cancer clinics in the same study area had already established that 87% of patients made the journey by car and that travel estimates based on the road network and average speeds were closely related to actual car journey times reported by patients (Haynes *et al*, 2006).

With the exception of the thoracic surgery analysis, the institution chosen for analysis of access was the closest NHS facility that provided services for diagnosis of lung cancer, that is, the closest facility to which the GP might refer a patient for this purpose. A specialist hospital that provided non-surgical treatment but not diagnosis for cancer was excluded from this and some very small hospitals, attended by fewer than 1% of the patients, and private healthcare providers, which were attended by fewer than 400 patients in the database, were also excluded.

We explored the characteristics of the closest hospital as determinants for receipt of chemotherapy, comparing designated cancer centres with district general hospitals that hosted a non-surgical oncology service and those that did not.

Deprivation was determined from the Index of Multiple Deprivation (IMD), an area-level measure associated with the postcode (Office of the Deputy Prime Minister, 2004). We removed the access to services domain from the IMD scores so as to eliminate the potential of double counting.

Patients were divided into equal quartiles for deprivation and for travel time to the closest hospital providing diagnostic access. All statistical analyses were undertaken using the SPSS for Windows 14 package (2005, SPSS Inc., Chicago, IL, USA). Binary logistic regression was used to examine the relationship between the odds of undergoing an intervention and deprivation and travel time. Models were adjusted for the effects of patient age and sex (Patel *et al*, 2007, Jones *et al*, 2008a, 2008b). The stage of the disease was not recorded in the NYCRIS data set. It is likely that this patient feature has a causal effect in patients being denied thoracic surgical treatment. The likelihood of undergoing this was determined for 18 324 non-small cell patients. For small-cell lung cancer patients (*n*=5510) likelihood of receiving chemotherapy was determined. The intent, curative or palliative could not be determined from this database.

## Results

Of the 34 923 patients, 26 069 (74.6%) had the diagnosis of lung cancer confirmed histologically. 8816 (25.2%) did not have a histological diagnosis and for 38 (0.1%) no information regarding histological diagnosis was available. Just 1.1% of the sample attended a private healthcare provider for initial hospital treatment. Some 5783 (16.6%) received chemotherapy; 13 857 (39.7%) received radiotherapy and 3552 (10.2%) underwent surgery to the thorax. In total, 19 667 (56.3%) received at least one of these treatments. In this study, 3335 (18.2%) of the 18 324 non-small cell lung cancer patients underwent thoracic surgery. Then, 3619 (65.7%) of the 5510 small-cell lung cancer patients received chemotherapy.

Table 1 shows the distribution of the 34 923 patients according to deprivation and travel quartiles. Baseline categories were travel quartile 1, which represents the shortest travel time to hospital, and deprivation quartile 1, which represents the least deprived. The 25th percentile travel time was 7.00 min, the median 10.91 min and the 75th percentile 15.49 min. In the study area the majority of hospitals are located in urban centres and the proportion of the most deprived patients was greatest in the closest travel quartile and least in the furthest.

For histological confirmation of diagnosis (Table 2), there was a strong trend for patients living in more deprived areas to be less likely to be confirmed. Although there was no effect of travel time overall, the magnitude of the trend with deprivation tended to increase with increasing travel time so the lowest odds of confirmation was observed among those residing in the most deprived and furthest travel time quartile. Patients in the intermediate groups for deprivation and travel time did not differ greatly in the rate of histological confirmation from the reference group, and the odds ratios did not reach statistical significance.

For receipt of any active treatment, that is surgery, radiotherapy or chemotherapy, the decrease in receipt of treatment with increasing deprivation was once more apparent with the strongest evidence of distance decay, that is the tendency for utilisation of a service to decline with increasing distance between the patient's residence and its location, again being evident for the most deprived group living at the longest travel times (Table 3).

Table 4 shows the odds of undergoing thoracic surgery, the definitive optimal treatment for non-small cell lung cancer, in relation to deprivation and travel time firstly to the nearest hospital providing diagnostic services (Table 4a) and also offering this treatment (Table 4b). There was again a tendency for the more deprived patients, and again the effect was greatest for those in the most deprived quartile and furthest from hospital, irrespective of whether this was the closest diagnosing hospital or the closest thoracic surgery unit. Patients whose closest NHS diagnostic unit was a District General Hospital (DGH), regardless of whether there was a medical oncology service present, were significantly less likely to undergo thoracic surgery compared with those whose closest hospital was a cancer centre. (DGH with oncology, OR=0.87, 95% CI=0.76–0.99, *P*=0.036; DGH without oncology, OR=0.88, 95% CI=0.80–0.96, *P*=0.005, all adjusted for age and sex)

For administration of chemotherapy to patients with small-cell lung cancer (the optimal treatment in such patients), there was no consistent variation with social deprivation or with distance (Table 5) and, while the lowest odds of receiving the treatment was again among those in the most deprived and furthest quartile, this did not reach statistical significance. When such patients attended a DGH which included oncology services, usually medical oncology, the odds ratio for receiving chemotherapy compared with patients attending a designated cancer centre was 0.94 (95% CI 0.77–1.16); those attending a District General Hospital without oncology were less likely to receive this treatment (OR 0.78, 95% CI 0.68–0.90).

## Discussion

In this study, we found that lung cancer patients living in the most deprived areas were the least likely to receive histological diagnosis, active treatment and thoracic surgery for non-small cell cancer. Although there was no overall effect of travel time to hospital across the sample of patients, travel time was found to amplify the effects of deprivation, with the lowest likelihood of receiving these treatments falling within the most distant and deprived quartile.

The IMD 2004 score on which this is based is allocated to a Lower Level Super Output Area (SOA) with a population of approximately 1500 (Office of the Deputy Prime Minister, 2004). The relevant SOA for an individual patient is identified from the postcode. While there is inevitably variation in socioeconomic circumstances between individuals in that population the effect of this on the interpretation we have given to these data will be to diminish the differences we have observed if favoured residents of a deprived SOA increase the intervention rate and deprived residents of an affluent SOA decrease it.

The consistent observation that patients with lung cancer fare worse in the United Kingdom than they do in socioeconomic peer group nations of Western Europe requires careful scrutiny. The data collected by cancer registries enable this observation to be made and in this analysis we use these data to explore socioeconomic influences in relation to the geography of healthcare provision. The developing process of detailed collection of data about the management of individuals takes the study further. The recently published National Lung Cancer Audit for England and Wales has shown a similar pattern to previous analyses in spite of the development and refinement of referral guidelines for people with suspected cancer. Of patients accrued into the 2006 audit, only 48% had been referred as requiring investigations for lung cancer by their GP (National Lung Cancer Audit, 2007). Before the guidance was published, the figure was virtually the same (Melling *et al*, 2002). The proportion of patients undergoing thoracic surgery and other specific treatments was low compared with other West European countries.

Travel times were based on the nearest appropriate hospital, which is not necessarily the same as the hospital to which each patient was actually referred. Although 94% of patients who received radiotherapy and 72% of patients who received thoracic surgery in our sample were treated at their nearest facility, only 44% of patients received chemotherapy at their closest unit. We assume that the patients who are willing to travel further to be treated at a preferred hospital are generally not those with transport difficulties. It was necessary to use the closest unit for comparative purposes because there was no hospital of actual referral for patients who were not treated. Furthermore, this approach eliminates any bias caused by a GP referring fitter patients to a specialist service while unfit individuals are seen locally.

The indicators we chose are the same as those included in the National Audit because they are those where there is variation. Our work may be criticised because our patients were diagnosed before the full implementation of the reforms of cancer services that have taken place in the United Kingdom over the past 8 years. Our response is that these changes concerned the organisation and practice in secondary care facilities, principally hospitals and had no influence on events before the involvement of the specialist team. The differences in indicators associated with deprivation associated with the immediate area of residence of the patient and the travel time to the closest healthcare institution with the facilities to make the diagnosis of lung cancer are more likely to reflect factors affecting access to the hospital, not what happens within it.

In investigating the effect of these we add detail to the smaller studies conducted in Scotland which showed that patients with lung cancer were diagnosed with more advanced disease with increasing distance of a cancer centre from their residence and that independently of age, stage and other factors deprived people were less likely to receive chemotherapy (Campbell *et al*, 2001, 2002).

The NYCRIS data set contains virtually no information about stage at presentation. It is very likely that the disadvantage of socioeconomic deprivation and prolonged travel to services is manifest in more advanced stage at presentation. This would mainly affect the use of thoracic surgery, a treatment, which is reserved for patients with disease confined to structures that can be excised. However, histological diagnosis is an important diagnostic step in all patients so the stage of disease is not expected to influence its attainment. Where surgery is not possible, radiotherapy or chemotherapy may be offered unless contraindicated by comorbidity or poor performance status so stage of disease is not likely to influence the likelihood of being denied any treatment as outlined in Table 3.

The general finding for active treatment is that deprivation is strongly associated with reduced delivery and for the most deprived this is exacerbated by distance between residence and hospital. It is most likely that this relates to the events before attendance at the hospital, especially the involvement of primary care in attaining the diagnosis of a tumour. The confirmation of the histological diagnosis is used as a marker of the starting point for active management; this may not be attained if patients are felt to be too unfit for investigative tests, which raises the question of what opportunities were missed in primary care. The findings of Hamilton *et al* (2005), derived from primary care studies in the city of Exeter, have shown the tendency for patients ultimately diagnosed with lung cancer to have a record of attendances with relevant symptoms over several months preceding the diagnosis.

The fact that the effects were less for SCLC chemotherapy is likely to reflect the fact that this type of the disease is rapidly progressive so that a relatively high proportion of patients who in fact had SCLC were considered too unwell for diagnostic procedures and so are never identified.

The lack of distance decay in the histological confirmation rate for patients of intermediate deprivation suggests that travel to hospital was less of a barrier for people from average socioeconomic backgrounds than it was for the group from the most deprived areas. An explanation of this might be that the closest quartile is affected by the problems of delivery of primary care services in an inner city area, most hospitals are located in urban areas. This appears to be less important in suburban areas with other issues of access becoming more important in the furthest, most rural travel quartile. The questions raised by the analysis that we report need to be explored by detailed studies of access to lung cancer services in primary care. Differences in uptake of treatment have also been demonstrated in south east England (Jack *et al*, 2003).

Because thoracic surgery was provided in a small number of hospitals with more patients therefore having to travel further, a clear distance decay effect was apparent with the patients living in the furthest travel quartile being the least likely to undergo such treatment. In a previous analysis of these data we showed that for all lung cancer patients there was a distance decay effect for access to thoracic surgery (Jones *et al*, 2008b); this included access both to operative treatment and surgical diagnostic tests. The present analysis shows independent effects of both travel time and social deprivation in the sub-group of patients who have histologically confirmed non-small cell lung cancer.

Where healthcare provision is mostly provided by private insurance, as in the USA, it has been shown that lack of such insurance is associated with more advanced stage at presentation (Halpern *et al*, 2008). These data show that deprivation is very important even when healthcare is provided free of charge.

Active treatment is associated with initial referral to a specialist in lung cancer management (Jack *et al*, 2006). It is not possible in this study to assess the effectiveness or otherwise of DGHs in providing services, although there is clear evidence that patients are more likely to receive chemotherapy, the definitive treatment for small-cell lung cancer, if their closest hospital is either a cancer centre or a DGH at which a non-surgical oncologist is based. Patients whose closest diagnostic hospital was a DGH were significantly less likely to undergo thoracic surgery compared with those whose closest hospital was a cancer centre.

In planning services, it is necessary to make available appropriate financial resources. There is strong evidence in these data that by the attainment of a high rate of histological diagnosis and higher rates of treatment in general and thoracic surgery in particular, patients from the least deprived areas make the most use of resources. In the NHS funding systems are weighted to direct resources to meet the undeniable healthcare needs of deprived people, but the increased consumption of resources by people who are from affluent areas in the entirely appropriate pursuit of diagnosis and treatment of lung cancer represents an increased call on the relatively lower level of funding for their areas of residence. This should be considered as one explanation for the reported financial stress in the NHS organisations responsible for such areas. To correct the geographical and especially social inequities that we have shown, the NHS will probably require extra capacity to meet the demand this will generate. It seems that patients from more socioeconomically deprived areas could be further disadvantaged if centralisation of services increased their travel time to access them.

## Conclusion

Attainment of histological diagnosis and receipt of active treatment are strongly influenced by socioeconomic deprivation. For the most deprived groups, disadvantage is amplified among those with increased travel time to services. Further development of NHS lung cancer services should therefore be directed at overcoming both of these adverse influences on uptake.

## Figures and Tables

**Table 1:** Number of patients (%) by quartiles of deprivation and travel time to the closest hospital providing diagnostic services

**Deprivation**					
**Travel time**	**1 Least**	**2**	**3**	**4 Most**	**All quartiles**
1. Closest	1134 (3.2)	1976 (5.7)	2181 (6.2)	3465 (9.9)	8756
2.	1579 (4.5)	2186 (6.3)	2365 (6.8)	2587 (7.4)	8717
3.	2104 (6.0)	2379 (6.8)	2502 (7.2)	1737 (5.0)	8722
4. Furthest	3915 (11.2)	2193 (6.3)	1683 (4.8)	937 (2.7)	8728
All quartiles	8732	8734	8731	8726	Total
					34923

**Table 2:** Odds ratios (95% CI) for attaining histological diagnosis by quartiles of deprivation and travel time. All values adjusted for age and sex

**Deprivation**					
**Travel time**	**1 Least**	**2**	**3**	**4 Most**	**All quartiles**
1. Closest	1	0.79^*^ (0.66–0.94)	0.69^**^ (0.58–0.82)	0.74^**^ (0.62–0.87)	1
2.	0.88 (0.73–1.07)	0.81^*^ (0.67–0.96)	0.80^*^ (0.67–0.95)	0.75^**^ (0.63–0.89)	1.05 (0.97–1.13)
3.	0.90 (0.76–1.08)	0.87 (0.73–1.04)	0.87 (0.73–1.04)	0.78^**^ (0.65–0.94)	1.13^**^ (1.05–1.21)
4. Furthest	0.83^*^ (0.70–0.97)	0.78^**^ (0.65–0.93)	0.78^**^ (0.65–0.94)	0.61^**^ (0.49–0.75)	1.02 (0.95–1.09)
All quartiles	1	0.93^*^ (0.86–1.00)	0.90^**^ (0.83–0.96)	0.84^**^ (0.78–0.90)	

^*^ *P*<0.05, ^**^ *P*<0.01.

**Table 3:** Odds ratios (95% CI) for receipt of any active treatment by quartiles of deprivation and travel time. All values adjusted for age and sex

**Deprivation**					
**Travel time**	**1 Least**	**2**	**3**	**4 Most**	**All quartiles**
1. Closest	1	0.85^*^ (0.73–1.00)	0.76^**^ (0.65–0.89)	0.74^**^ (0.64–0.86)	1
2.	0.91 (0.77–1.07)	0.85^*^ (0.72–0.99)	0.76^**^ (0.65–0.88)	0.74^**^ (0.64–0.86)	1.00 (0.93–1.06)
3.	0.89 (0.76–1.04)	0.84^*^ (0.72– 0.98)	0.80^**^ (0.69–0.93)	0.78^**^ (0.66–0.92)	1.03 (0.97–1.10)
4. Furthest	0.93 (0.80–1.07)	0.83^*^ (0.71–0.97)	0.71^**^ (0.60–0.83)	0.55^**^ (0.46–0.67)	1.01 (0.95–1.08)
All quartiles	1	0.91^**^ (0.86–0.97)	0.82^**^ (0.77–0.88)	0.79^**^ (0.74–0.84)	

^*^ *P*<0.05, ^**^ *P*<0.01.

**Table 4:** Odds ratios (95% CI) of undergoing thoracic surgery for non-small cell lung cancer (*n*=18 324 patients) by quartiles of deprivation and travel time to (a) the closest diagnosing hospital and (b) the closest thoracic surgery unit. All values adjusted for age and sex

**Deprivation**					
**Travel time**	**1 Least**	**2**	**3**	**4 Most**	**All quartiles**
*(a)*					
1. Closest	1	0.87 (0.67–1.12)	0.72^*^ (0.56–0.93)	0.75^*^ (0.59–0.94)	1
2.	0.99 (0.76–1.29)	0.78 (0.60–1.00)	0.85 (0.67–1.09)	0. 69^**^ (0.54–0.88)	1.01 (0.91–1.12)
3.	0.87 (0.68–1.12)	0.84 (0.65–1.07)	0.75^*^ (0.58–0.96)	0.86 (0.66–1.11)	1.03 (0.93–1.15)
4. Furthest	0.88 (0.70–1.11)	0.81 (0.63–1.04)	0.65^**^ (0.49–0.85)	0.60^**^ (0.44–0.84)	0.99 (0.89–1.10)
All quartiles	1	0.90^*^ (0.81–1.00)	0.82^**^ (0.74–0.91)	0.80^**^ (0.72–0.89)	
					
*(b)*					
1. Closest	1	0.91 (0.69–1.19)	0.80 (0.61–1.04)	0.70^**^ (0.55–0.89)	1
2.	0.86 (0.65–1.13)	0.81 (0.62–1.05)	0.71^**^ (0.54–0.92)	0. 75^*^ (0.58–0.98)	0.98 (0.88–1.09)
3.	0.79 (0.61–1.02)	0.65^**^ (0.50–0.85)	0.64^**^ (0.49–0.83)	0.61^**^ (0.46–0.81)	0.86^**^ (0.77–0.95)
4. Furthest	0.83 (0.65–1.06)	0.71^*^ (0.54–0.92)	0.62^**^ (0.46–0.82)	0.55^**^ (0.39–0.76)	0.91 (0.82–1.01)
All quartiles	1	0.90^*^ (0.81–1.00)	0.82^**^ (0.74–0.91)	0.80^**^ (0.72–0.89)	

All values adjusted for age and sex. ^*^ *P*<0.05, ^**^ *P*<0.01.

**Table 5:** Odds ratio (95% CI) of receiving chemotherapy for small cell lung cancer (*n*=5510 patients) by quartiles of deprivation and travel time. All values adjusted for age and sex

**Deprivation**					
**Travel time**	**1 Least**	**2**	**3**	**4 Most**	**All quartiles**
1. Shortest	1	1.24 (0.83–1.85)	0.92 (0.63–1.35)	1.21 (0.85–1.74)	1
2.	1.23 (0.81–1.87)	1.40 (0.95–2.06)	1.11 (0.76–1.62)	1.29 (0.88–1.89)	1.14 (0.96–1.34)
3.	1.49^*^ (1.01–2.21)	1.50^*^ (1.03–2.20)	1.45 (0.99–2.12)	1.31 (0.87–1.98)	1.31^**^ (1.11–1.55)
4. Longest	1.27 (0.89–1.82)	1.45 (0.98–2.16)	1.25 (0.83–1.89)	0.81 (0.52–1.28)	1.12 (0.95–1.32)
All quartiles	1	1.10 (0.94–1.30)	0.91 (0.78–1.08)	0.94 (0.80–1.11)	

^*^ *P*<0.05, ^**^ *P*<0.01.
